# Interview with the 2018 *JDB* Travel Award Winner

**DOI:** 10.3390/jdb6020010

**Published:** 2018-05-05

**Authors:** Journal of Developmental Biology (JDB) Editorial Office

**Affiliations:** MDPI, St. Alban-Anlage 66, 4052 Basel, Switzerland; jdb@mdpi.com

The winner of the 2018 *JDB* Travel Award was granted to Ms. Victoria Deneke, BS, who is a fifth-year graduate student in Dr. Stefano Di Talia’s laboratory in the Department of Cell Biology at Duke University Medical Center, USA. Ms. Deneke was supported with 800 Swiss Francs towards travel expenses to attend the 2018 EMBO/EMBL Symposium on “Tissue Self-Organization: Challenging the Systems”.

Victoria is using *Drosophila* as her model organism system and addressing the mechanisms of synchronization of mitosis in the earlier phases of embryonic development, thereby ensuring coordinated normal development. She has taken an interdisciplinary approach, combining molecular cell biology and embryology with quantitative live imaging and mathematical modelling, and was able to demonstrate in an elegant *Developmental Cell* paper [1] that waves of Cdk1 activity synchronize the cell cycle in early *Drosophila* embryos. These studies provide a new mechanism by which global synchronization can arise from a spreading of local synchrony. She has received several honours and awards, including a HHMI International Student Research Fellowship, a Schlumberger Faculty for the Future Fellowship and was a Notre Dame Hesburgh International Scholar.

In this interview, we would like to find out more about the growing path of her academic career.

**1. We noticed your Bachelor’s Degree was in Chemical Engineering. Why did you choose Cell and Developmental Biology for PhD study?**

I have always held an appreciation for the biological sciences and while studying Chemical Engineering at the University of Notre Dame, I found a research position in an ovarian cancer lab. This research experience provoked my interest in interdisciplinary work, and prepared me to venture into the biomedical sciences at Duke University for my PhD. While transitioning to Graduate School, I desired a lab that would coincide with my interdisciplinary background. I joined Dr. Stefano Di Talia’s research group (https://sites.duke.edu/ditalialab/, Figure 1), which integrates principles from physics, mathematics, and biology to uncover the signaling pathway dynamics that control cell division during development and regeneration. I have thoroughly enjoyed doing research in Cell and Developmental Biology as an interdisciplinary scientist. As we keep uncovering fundamental principles in biology, it will become more and more important to bring together scientists from different fields to solve our greatest scientific challenges.

**2. Could you please briefly describe the research you are currently working on? What is the significance of this research?**

I study the first hours of development in fruit fly embryos. Fruit fly eggs are fascinating because they develop remarkably fast and in a synchronous manner. Therefore, we have used this model to learn how biological cells can communicate in fast timescales. My first project consisted of figuring out how the early divisions are coordinated in the early fly embryo. We discovered that these nuclear divisions are synchronized through chemical waves of Cdk1, one of the main drivers of mitosis in cells [1]. We have also uncovered that these waves arise through a novel physical mechanism, which generates chemical waves in biological systems that exhibit transient bistability [2]. Our studies have added to the numerous new examples that further illustrate the importance of chemical waves as an essential means of communication in biological systems [3]. My current project delves into the mechanism by which such synchrony arises, which involves the precise spatiotemporal integration of mechanical and biochemical pathways. This work is significant because we are studying mechanisms of communication in cells that, when dysfunctional, can lead to health disparities and disease.

**3. How was your overall experience at the conference you attended? Did you have a chance to expand your professional network as you’d hoped?**

The “2018 Tissue Self-Organisation: Challenging the Systems” Conference at EMBL Heidelberg was a phenomenal scientific and professional experience. This meeting brought together scientists from a wide range of fields interested in mechanisms of self-organization in biological systems. I was able to interact with many young and established scientists, and receive feedback on my science. I would highly recommend this meeting to researchers interested in interdisciplinary work integrating mechanics, mathematical modelling, organogenesis, and collective cell behavior.

**4. While making successful progress in your research projects, you have also participated in many social activities. How do you manage your time?**

I think one should invest time in activities that one particularly enjoys. In my case, outside of research, I love dancing and building communities. I have made sure to immerse myself in graduate student groups, like Duke INSPIRE and Duke BioCoRE, that have enabled me to be part of a supportive community that brings people together to discuss science or have conversations on the scientific diversity. I have also attended a weekly salsa class in a Durham studio for the last couple of years and enjoy going out dancing with friends.

**5. What are your future plans? Where do you see yourself in five years?**

I would like to continue doing research in quantitative biology as a postdoctoral scientist. In the long run, I see myself as an interdisciplinary scientist in an academic environment addressing questions in cell and developmental biology. There are many exciting questions in biology that will keep us busy for many decades to come.

**6. You have published one paper in an open access journal. What is your opinion on how open access contributes to the publishing world?**

We recently held a discussion in one of our student clubs called Duke INSPIRE on open-access journals and the future of science communication. I am in full support of open access journals, which are changing the way that science is communicated to the world and are facilitating scientific exchange. As science becomes more accessible, it will be imperative to develop our communication skills as a scientific community so that our findings are distributed and used appropriately.

## Figures and Tables

**Figure 1:**
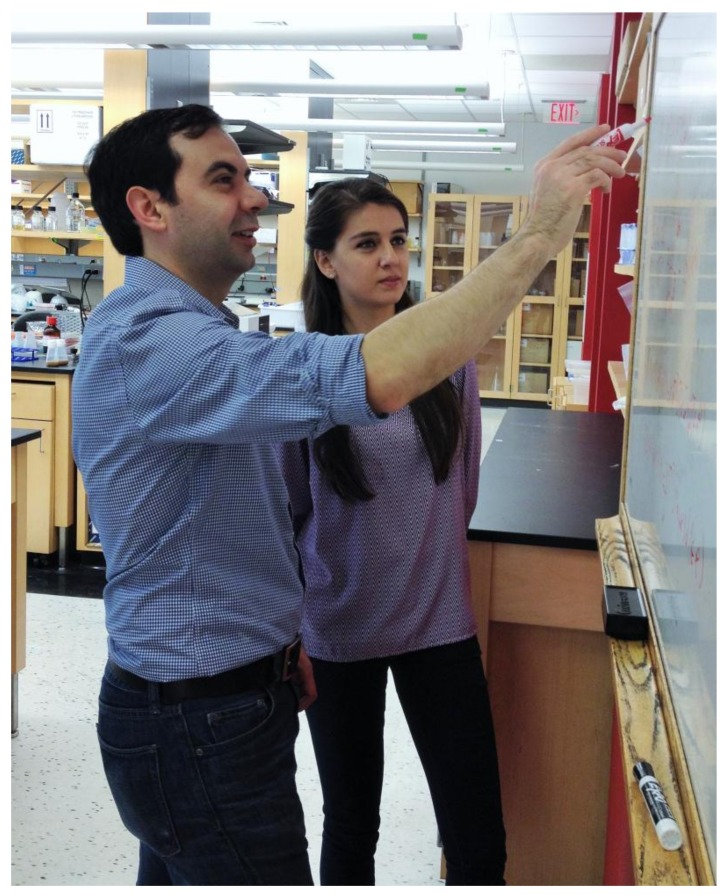
Victoria is discussing with her mentor Dr. Stefano Di Talia.
